# The Application of Multimodal Data Fusion Algorithm MULTINet in Postoperative Risk Assessment of TAVR

**DOI:** 10.3390/jcm14248620

**Published:** 2025-12-05

**Authors:** Wei He, Jiawei Luo, Xiaoyan Yang

**Affiliations:** 1 West China Biomedical Big Data Center, West China Hospital/West China School of Medicine, Sichuan University, No. 37 Guoxue Xiang, Chengdu 610041, China; 2023224020620@stu.scu.edu.cn (W.H.); jiaweiluo@wchscu.cn (J.L.); 2 Med-X Center for Informatics, Sichuan University, No. 37 Guoxue Xiang, Chengdu 610041, China

**Keywords:** TAVR, mortality, risk, prediction, multimodal data, MULTINet (multimodal learning for TAVR risk network)

## Abstract

**Background:** Transcatheter aortic valve replacement (TAVR) has emerged as a pivotal minimally invasive interventional therapy for aortic valve disease and has seen increasingly widespread clinical adoption in recent years. Despite its overall safety, the adverse events and even deaths in the postoperative period still account for a certain percentage. Accurate identification of high-risk patients is therefore critical for optimizing preoperative decision making, guiding individualized treatment strategies and improving long-term outcomes. However, existing scoring systems and predictive models fail to fully leverage multimodal clinical data from patients, resulting in suboptimal predictive accuracy that falls short of the demands of precision medicine, indicating substantial room for improvement. **Methods:** In this study, a multimodal deep learning model named MULTINet (multimodal learning for TAVR risk network) was constructed using data from the MIMIC-IV (Medical Information Mart for Intensive Care) cohort. This model achieved unimodal and multimodal modeling through a dual-branch structure, and, by using an attention pooling fusion module, flexibly handled the input that contained missing modalities, to predict the 30-day all-cause mortality in TAVR patients. The area under the receiver operating characteristic curve (AUC), the area under the precision–recall curve (AUPR) and the recall rate were used for prediction evaluation. The calibration degree was evaluated by calibration diagrams and Brier scores, and its clinical practicability was assessed through decision curve analysis (DCA). And the integrated gradient method was used to identify key predictive features to enhance interpretability of the model. **Results:** In the postoperative 30-day all-cause mortality prediction task, the MULTINet method achieved an AUC value of 0.9153, AUPR value of 0.5708 and Recall value of 0.8051, which was significantly superior to the XGBoost method (AUC 0.8958, AUPR 0.4053 and Recall 0.7793) and the MedFuse method (AUC 0.5571, AUPR 0.2487 and Recall 0.3089). The MULTINet method demonstrated more robust and reliable probability estimation performance, with a Brier score of 0.0269, outperforming XGBoost (0.0343) and MedFuse (0.2496). It achieved a higher net benefit in decision analysis, reflecting its effectiveness in strategy optimization and actual decision-making benefits. The renal function, cardiac function and inflammation-related indicators contributed greatly in the prediction process. **Conclusions:** The multimodal deep learning model proposed in this study named MULTINet enables adaptive integration of multimodal clinical information for predicting all-cause mortality within 30 days post-TAVR, substantially improving both predictive accuracy and clinical applicability, providing robust support for clinical decision making and boosting TAVR management toward greater precision and intelligence.

## 1. Introduction

Recently, transcatheter aortic valve replacement (TAVR) has developed rapidly in the field of cardiovascular surgery and has become an innovative treatment technique for aortic valve diseases. With continuous advancements in device design and imaging technologies, TAVR has been increasingly adopted and standardized around 2010, offering new therapeutic options for patients with aortic stenosis who were previously considered inoperable or at high risk for surgical intervention [1]. TAVR not only significantly reduces the surgical trauma and pain for patients but also shortens the hospital stay and lowers the incidence of some complications [2]. Among patients with severe aortic valve stenosis at low risk, TAVR is superior to surgical aortic valve replacement (SAVR) in terms of all-cause mortality or disabling stroke at three-year follow up [3].

The existing traditional risk scoring systems, such as the Society of Thoracic Surgeons Predicted Risk of Mortality (PROM) score [4] and the European System for Cardiac Operative Risk Evaluation II (EuroSCORE II) [5], have limited discriminatory capabilities in TAVR patients [6], encouraging researchers to apply machine learning (ML) and deep learning (DL) methods for individualized death risk prediction. The risk prediction models of artificial intelligence methods provided more accurate risk assessment results based on the relevant data of patients, thereby improving clinical decision making [7,8,9,10,11].

However, patient information is inherently multimodal including electronic health records (including demographics, medical history, and laboratory tests), preoperative and intraoperative imaging, intraoperative monitoring data, and even genomic/omics profiles, all of which contain information relevant to postoperative outcomes. Evidence has shown that deep integration of imaging and structured clinical data can markedly enhance predictive performance [12]. Multimodal learning thus represents a powerful approach to improving the performance of clinical prediction models [13]. Nevertheless, comprehensive acquisition of multimodal data for every patient remains difficult to achieve in routine clinical practice. Data missingness is pervasive and highly heterogeneous [14], often associated with patients’ clinical characteristics, socioeconomic status, or physicians’ decision making. If model development naively assumes modality completeness at both training and deployment stages, the model will be difficult to adapt to the changes and uncertainties in real scenarios, thereby being significantly limited in terms of generalization ability and robustness. Therefore, it is particularly crucial to construct a clinical prediction model that can robustly handle missing modalities [15].

Current studies have made preliminary efforts to predict the postoperative outcomes of TAVR by leveraging patients’ multimodal data—including imaging studies, electronic health records, and laboratory test results. However, most of these investigations still depend on clinical experts to manually extract features [16]. A key advantage of this approach is its high interpretability, which helps doctors understand the rationale behind the model’s decisions and thus bolsters clinical trust. Nevertheless, manual feature extraction suffers from several drawbacks, especially when dealing with unstructured data, which is particularly cumbersome, time-consuming, and prone to introducing human bias [17]. Currently, there is no model that integrates complete multimodal information such as structured time series, original electrocardiograms (ECGs), and clinical text annotations at the same time.

Accordingly, this study aims to develop a multimodal deep learning model, which, in the real clinical environment, integrates multimodal data of laboratory tests, temporal monitoring data, electrocardiogram signals and clinical text information and is capable of adaptively handling modality missingness, with the goal of improving both the accuracy and clinical interpretability of 30-day mortality prediction following transcatheter aortic valve replacement (TAVR), thus achieving a more robust assessment of the TAVR prognosis.

## 2. Materials and Methods

### 2.1. Dataset

The publicly available MIMIC-IV database [18] was used as the study cohort. MIMIC-IV is an openly accessible, de-identified critical-care repository that integrates clinical data from patients admitted to Beth Israel Deaconess Medical Center between 2008 and 2019. It encompasses a broad spectrum of information, including physiological monitoring, therapeutic interventions, medication usage, laboratory tests, imaging reports, and nursing notes. The patient records related to TAVR surgery were screened from the “proceres_icd” program sheet through ICD (International Classification of Diseases) -9 and ICD-10 codes. The diagnosis and treatment data of the patients were included after excluding those with repeated hospitalizations and repeated surgeries. Each patient was uniquely identified by a “subject_id” identifier. According to the patient identifier, the most recent multimodal diagnosis and treatment data of patients before TAVR were selected, including demographic characteristics, laboratory test results, ICU time-series monitoring data, electrocardiograms (ECGs) with their accompanying machine-generated annotations, and clinical text annotations.

### 2.2. Data Preprocessing

Structured data comprised demographic information, laboratory test results, and ICU monitoring data. Laboratory test features were constructed using the most recent preoperative measurement as the representative value; missing values were imputed using the Multiple Imputation by Chained Equations (MICE) method [19]. MICE method constructs a series of regression models based on the observed information in the dataset to iteratively model and impute each missing variable, ultimately generating multiple complete datasets. These datasets are then combined via Rubin’s rules [20], yielding more robust estimates that account for the uncertainty introduced by missingness. MICE method enables effective imputation of missing values in complex multivariable datasets and has been widely employed in epidemiological research [21]. ICU time-series monitoring data exhibited inconsistent observation time points, varying sampling frequencies, and missing values, which posed challenges for direct modeling. To convert these asynchronous, non-uniformly sampled dynamic variables into a structured representation on a uniform time scale, the average time interval ${\bar{\Delta }}_{j}$ between successive observations was calculated for each monitoring variable across patients, and all time points were then aligned by resampling. Let $x_{i,j,k}$ denote the *k*-th observation of the *j*-th examination item for the *i*-th patient and its corresponding timestamp, with the time interval between two adjacent observation points being $\Delta_{i,j,k}=t_{i,j,k+1}-t_{i,j,k}$, the average value ${\bar{\Delta }}_{j}$ of the observation intervals of all patients on the monitoring *j*-th item could be obtained, which is given by Equation (1):

(1) $${\bar{\Delta }}_{j}=\frac{1}{\sum_{i=1}^{N}(K_{i,j}-1)}\sum_{i=1}^{N}\sum_{k=1}^{K_{i,j}-1}\Delta_{i,j,k}$$

where $K_{i,j}$ denotes the number of the *j*-th examination item monitoring point of the *i*-th patient, and N denotes the number of patients. For the *j*-th monitoring item of the *i*-th patient, the original observation sequence ${\left\{(t_{i,j,k},x_{i,j,k})\right\}}_{k=1}^{K_{i,j}}$ was aligned onto this temporal grid by resampling and aggregation. Given a resampling time point $t_{l}$, there are multiple observations $\left\{x_{i,j,k}\|t_{i,j,k}-t_{l}|\leq \delta \right\}$ within a preset time window half-width δ. Different aggregation functions were used for different variable types, such as mean for continuous monitoring values and mode for discrete values. If there were missing values for this observation, the first missing value was filled in using Last Observation Carried Forward (LOCF), other missing numeric variables were filled in using linear interpolation, and discrete variables were filled in using null values.

Unstructured data included electrocardiograms (ECGs) with their accompanying machine-generated annotations and clinicians’ narrative reports from imaging studies such as Computed Tomography (CT) or Magnetic Resonance Imaging (MRI). The MIMIC-IV database provides raw 12-lead ECG recordings sampled at 500 Hz over 10 s. For each diagnostic ECG, the machine also produces a summary report and key waveform metrics (e.g., RR intervals, QRS onset and offset, etc.). For each patient, the last preoperative examination result of the ECG is retained as the input data. If the ECG data is abnormal and there is clean ECG data from the previous time, it will be replaced. For the waveform data of each electrocardiogram, ECG-FM [22] was used for preprocessing. ECG-FM (Electrocardiogram Foundation Model) adopts a Wav2Vec 2.0-inspired architecture and is pretrained via objectives such as masked signal modeling and contrastive learning to learn generalizable feature representations from ECG signals. By leveraging large-scale unsupervised pretraining and transfer learning, ECG-FM enhances performance and generalization in ECG analysis. By freezing the pre-trained ECG-FM encoder parameters, the corresponding embedding representation was obtained by inputting ECG data. Similarly, for text annotation data, Clinical ModernBERT [23] was used to extract context representation information to obtain the corresponding text embedding representation.

### 2.3. Postoperative 30-Day All-Cause Mortality Prediction for TAVR Based on Multimodal Data

**Figure 1:**
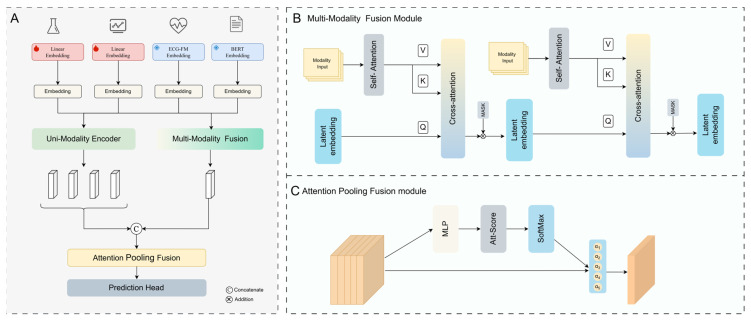
The framework of the MULTINet model: (**A**) The overall structural framework of the model. (**B**) Multi-Modality Fusion Module. Through learnable latent feature representations, the cross-attention mechanism is utilized to achieve intermodal interaction modeling. (**C**) Attention Pooling Fusion module. Attention scores are used to adaptively fuse different modal information. ECG-FM: electrocardiogram foundation model; MLP: multilayer perceptron; BERT: clinical modernbert; and Att-score: attention score, Q, K and V denote the query, key, and value embeddings, respectively.

Based on multimodal data, the proposed 30-day all-cause mortality prediction model for TAVR, that is, the MULTINet (multimodal learning for TAVR risk network), was divided into two modules, namely, the dual-branch module, which included a single-modal encoder and a multimodal fusion module, which processed the unimodal data and the data interaction among modalities, respectively. Then, the attention-based feature fusion mechanism was used to predict 30-day all-cause mortality after TAVR. The main framework of the model is shown in Figure 1. Let the multimodal input be $\left\{x_{l},x_{t},x_{e},x_{n}\right\}$, where $x_{l}$ are laboratory test results, $x_{t}$ are time-series measurements, $x_{e}$ are ECG waveforms, and $x_{n}$ are textual annotations. The laboratory and time-series inputs were each passed through learnable linear projections to produce embeddings that were aligned with the ECG and text embeddings. Due to the possible absence of input modalities, the multimodal fusion module mainly utilized a learnable latent feature representation. When the input modalities were missing, it compensated for the missing modal information through feature interaction based on the existing modal information. Given a multimodal combined embedding input $H_{comb}=[H_{l},H_{t},H_{e},H_{n}]$, where $H_{i}\in {\mathbb{R}}^{L_{i}\times D}$, the mask multi-head self-attention computed the information interaction within the modality via

(2) $$M\text{-}\mathrm{MHSA}(Q,K,V,M)=\mathrm{Softmax}(\frac{QW^{Q}{\left(KW^{K}\right)}^{T}}{\sqrt{d}}+M)VW^{V}$$

where Q, K, and V denote the query, key, and value embeddings, respectively; $W^{Q}$, $W^{K}$ and $W^{V}$ represent the corresponding weight matrix; M is the additional mask; and *d* represents the dimension of the embedding. The learnable latent feature representation is defined as $H_{latent}\in {\mathbb{R}}^{L\times D}$. Through the cross-attention mechanism, the learned latent feature representation is constantly updated with different modal inputs, defined as follows:

(3) $$\mathrm{M-MHCA}(H_{latent},K,V,M)=\mathrm{Softmax}(\frac{H_{latent}W^{Q}{\left(KW^{K}\right)}^{T}}{\sqrt{d}}+M)VW^{V}$$

where $H_{latent}$ represents the learnable query embedding, K and V represent key and value embeddings from different modal inputs, *d* represents the dimension of the embedding, and M is the additional input modal mask.

In order to deal with the missing situation in the multi-modal input data, a binary mask matrix $M_{u}$ was introduced to control the effectiveness of the feature update. Let the binary mask matrix be denoted as $M_{u}\in {\left\{0,1\right\}}^{B\times L\times D}$, where, if the *i*-th sample has missing modalities, the corresponding entries in the mask matrix for that sample are set to zero, effectively masking the subsequent computations and preventing updates at those positions. During batch training, the hidden state $H_{latent}\in {\mathbb{R}}^{L\times D}$ is expanded along the batch dimension to $H_{latent}\in {\mathbb{R}}^{B\times L\times D}$. For the (*t +* 1)-th update of $H_{latent}^{t+1}$, the M-MHCA mechanism was employed to perform cross-modal information interaction by utilizing the previous hidden state $H_{latent}^{t}$. The resulting representation is then element-wise multiplied by the mask matrix $M_{u}$ via the Hadamard product, thereby applying mask control to the updated process:

(4) $$H_{latent}^{t+1}=\mathrm{M-MHCA}(H_{latent}^{t},K,V,M)⊙M_{u}+H_{latent}^{t}$$

When a specific modality was missing for a given patient sample, the corresponding mask value was set to zero, thereby blocking that sample’s feature updates within the M-MHCA module. The module’s final output was obtained by adding the original feature vector to the mask-gated update, ensuring that—even in the presence of missing modalities—the model maintained stable information flow and representational capacity.

For the final fusion of different modality information, Attention Pooling Fusion mechanism was used to dynamically extract key information from the input, as shown in Figure 1C. In this module, a learnable linear layer assigned an attention weight to each modality’s embedding, while a masking mechanism suppressed invalid or missing modalities. The attention weights were normalized using SoftMax and regularized with Dropout. The final output was a weighted aggregation of the input sequence according to the attention weights, enabling the model to focus on key modality features while maintaining robustness to incomplete inputs.

The TAVR postoperative 30-day all-cause mortality prediction task involves dealing with highly imbalanced class distributions. In such scenarios, the commonly used cross-entropy loss function tends to bias the model towards optimizing performance on the majority classes, which have higher frequency. This results in the gradient updates being dominated by the majority class samples, thereby leading to poor recognition of the minority class samples at test time. Focal loss [24] addresses this by introducing a modulation term that down-weights the loss from easy examples and up-weights that from hard examples, thereby improving minority-class performance. However, merely increasing the loss contribution of difficult samples will lead to the model’s prediction being underconfident [25]. Therefore, Dual Focal Loss (DFL) [25] was used to adjust the loss contribution of simple samples and difficult samples by using the forecast confidence of the model. Based on DFL, a balance factor was added for balancing the weights of positive and negative samples by referring to the idea of Focal Loss, defined as follows:

(5) $$L_{\mathrm{DualFocal}}=-\alpha {\left(1-p_{k}+p_{j}\right)}^{\gamma }\cdot \log(p_{k})$$

where $p_{k}$ represents the probability of the true class, $p_{j}$ is the probability assigned to the other class, and the regulation factor γ controls the degree of modulation. By considering both the target and non-target class probabilities, DFL not only reduces the loss contribution from high-confidence predictions but also encourages the model to make more confident predictions by maximizing the gap between the target and non-target class probabilities. This helps to reduce the conservatism of the model while maintaining attention to difficult samples.

### 2.4. Experimental Setup

For hyperparameter settings, the embedding dimension of the overall model features was 768. Features of different modalities were projected to 768 dimensions through a fully connected network. The attention module had 8 heads and only uses a 1-layer transformer encoder network. Encoders of different modalities did not share parameters. The latent feature matrix that could be learned in the multimodal fusion module is 256 × 768, the model embedding dimension was also 768 dimensions, and the attention module had 8 heads. Dropout was uniformly set to 0.1, and LayerNorm was used for stable training. In the loss function, α was set to 0.25 and γ to 3. The Adam optimizer was used, with β_1_ = 0.9 and β_2_ = 0.999. The learning rate was initially set to 1 × 10^−4^. A total of 20 epochs were trained, and L2 regularization was employed to avoid overfitting. The dataset was trained using 5-fold cross-validation with a random seed of 2025 to obtain out-of-fold predictions, which were used to calculate the evaluation metrics, and the 95% confidence interval (95% CI) was estimated using bootstrapping with 1000 samples. Data processing and modeling were performed using Python (version 3.10.14), torch (version 2.4.0), matplotlib (version 3.9.1) and xgboost (version 3.0.2).

### 2.5. Model Evaluation

To evaluate the classification performance of the model for category-imbalanced data, two widely used evaluation metrics were adopted: the receiver operating characteristic curve (AUC) and the area under the precision–recall curve (AUPR). The AUC value illustrates the trade-off between the true positive rate (TPR) and the false positive rate (FPR) across different classification thresholds. The AUPR value, on the other hand, depicts the relationship between precision and recall at varying threshold settings. AUPR is particularly well-suited for scenarios with highly imbalanced class distributions. Furthermore, as this study focuses on the prediction of postoperative death cases (positive class samples), the recall rate (i.e., TPR) was adopted as the evaluation index, which could effectively reflect the model’s recognition ability for positive class samples and ensure that as many high-risk cases as possible were identified. The classification thresholds in different experiments are obtained by maximizing TPR-FPR through Youden Index. In addition, the Brier Score was also used to evaluate the accuracy of probability prediction. The relevant formulas are as follows:

(6) $$TPR=Recall=\frac{TP}{TP+FN}$$

(7) $$FPR=\frac{FP}{FP+TN}$$

(8) $$\mathrm{AUC}=\int_{0}^{1}TPR(FPR^{-1}(x))dx$$

(9) $$Precision=\frac{TP}{TP+FP}$$

(10) $$AUPR=\int_{0}^{1}Precision(Recall^{-1}(x))dx$$

(11) $$Brier=\frac{1}{N}\sum_{i=1}^{N}{\left(y_{i}-p_{i}\right)}^{2}$$

To comprehensively evaluate the performance of the constructed predictive model, in addition to the discrimination index, we further examined its calibration and clinical utility. Quantification was carried out, respectively, through calibration curve and decision curve analysis (DCA). Decision curve analysis quantified the net benefits of different prediction models under a series of clinically reasonable threshold probabilities. Net benefit considers both the benefits brought by true positives and the potential harm caused by false positives. Its calculation formula is

(12) $$\mathrm{Net}\;\mathrm{Benefit}(p_{t})=\frac{\mathrm{TP}(p_{t})}{N}-\frac{\mathrm{FP}(p_{t})}{N}\cdot \frac{p_{t}}{1-p_{t}}$$

### 2.6. Model Interpretability Analysis

To enhance the interpretability of the model’s predictions and to gain deeper insight into how input features influence the final decision, integrated gradients (IGs) [26] were adopted for interpretability analysis of the model. The IG method was utilized to conduct feature importance analysis on key prediction samples, identifying the input variables that have the greatest impact on model decisions. IG is a gradient-based attribution technique that apportions changes in the model’s output prediction to individual input features by integrating gradients along the straight-line path between the actual input and a chosen reference input. This method satisfies two crucial theoretical properties: Sensitivity and Implementation Invariance. For a given input x, the model function F produces the predicted probability for a particular class. Let $x^{\prime }$ be the reference input, which is usually set to the zero vector or task-specific baseline input, and define the interpolation input as follows:

(13) $$x_{\alpha }=x^{\prime }+\alpha (x-x^{\prime })$$

where α∈[0,1] represents the interpolation coefficient, then the integrated gradient attribution values $IG_{i}$ of different features $x_{i}$ can be defined as follows:

(14) $$IG_{i}(x)=(x_{i}-x_{i}^{\prime })\cdot \int_{\alpha =0}^{1}\frac{\partial F(x_{\alpha })}{\partial x_{i}}d\alpha$$

In practice, the above integral is usually estimated using numerical approximation methods, such as sampling m interpolation points uniformly:

(15) $$IG_{i}(x)\approx (x_{i}-x_{i}^{\prime })\cdot \sum_{k=1}^{m}\frac{\partial F(x_{\alpha_{k}})}{\partial x_{i}}\cdot \frac{1}{m}$$

where $\alpha_{k}=\frac{k-0.5}{m}$ represents the *k*-th interpolation step.

## 3. Results

To build the model, a total of 773 TAVR procedure-related patient records were screened by ICD-9 and ICD-10 codes. After excluding patients with repeated admissions and repeated procedures, 761 TAVR procedure records were included in the final analysis. The average age of the patients was 79.6 years (SD:9.33). There were 402 male patients (52.8%) and 359 female patients (47.2%). The number of patients who died within 30 days was 32 (4.2%).

The proposed MULTINet was compared with common machine learning algorithms. HAIM [13] has shown that, through extensive experiments, employing multimodal data fusion would effectively enhance performance in clinical health modeling. Following the HAIM framework, different pretrained feature-extraction models were used to process each modality and derive holistic patient representations, with decisions ultimately made by an XGBoost classifier. MedFuse [27] is an LSTM-based fusion model that integrates chest X-ray images and clinical time-series data to achieve effective in-hospital mortality prediction. Performance comparison experiments were conducted between the XGBoost model, the MedFuse model, and our method (MULTINet). Table 1 reports the performance of these methods on the MIMIC-IV multimodal dataset. The results indicated that our approach outperformed all compared methods, achieving an AUC of 0.9153 (95% CI: 0.87–0.95). Despite severe class imbalance, the proposed MULTINet method also attained an AUPR of 0.5708 (95% CI: 0.48–0.66), significantly exceeding that of the baselines. The recall rate of the model reached 0.8051 (95% CI: 0.71–0.90), indicating that it had strong sensitivity in identifying high-risk postoperative patients and was superior to the compared methods. The Brier score is 0.0269 (95% CI: 0.02–0.03), indicating that the model has reliable probability estimation performance.

**Figure 2:**
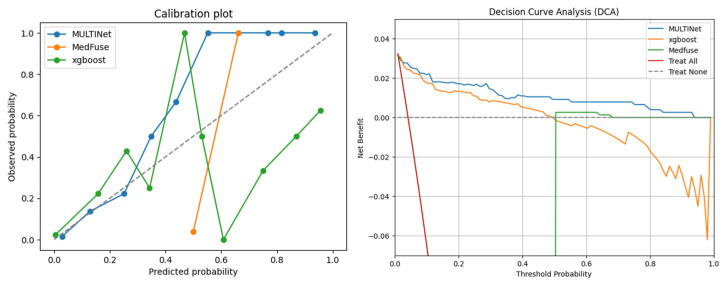
Calibration diagrams and decision curves of different models.

Calibration diagrams and decision curve analysis (DCA) were plotted to more comprehensively evaluate and compare the clinical practicability and predictive reliability of different models, as shown in Figure 2. Due to the significant changes in the net benefit of MedFuse, the decision curve has been appropriately scaled here. The detailed results are shown in Figure S1. As shown in the calibration diagrams (Figure 2, left), MULTINet exhibits the most reliable probability estimation across the full risk spectrum. Its calibration curve closely adheres to the ideal diagonal, indicating a good consistency between the predicted event rate and the observed event rate. In contrast, MedFuse shows a significant underestimation at lower prediction probabilities and a sharp overestimation at higher ranges, indicating calibration instability in sparse risk areas. The deviation of XGBoost is the largest, with significant oscillations and systematic calibration errors, especially in the medium to high predicted risk range. These results indicate that the model proposed in this paper is conducive to generating more stable and clinically interpretable probability outputs. Decision curve analysis (Figure 2, right) further supported the clinical utility of MULTINet. MULTINet consistently achieves the highest net benefit, outperforming MedFuse and XGBoost, as well as the “Treat All” and “Treat None” strategies. MedFuse’s performance drops rapidly outside the low threshold, while XGBoost shows significant volatility. As the threshold increases, its performance is inferior to that of the “no treatment” strategy. The stable and positive net benefit of MULTINet indicates that it is more suitable for risk-based decision-making scenarios, where accurately identifying high-risk patients is of crucial importance.

A comparison between uni-modality and multi-modality fusion methods was also performed. In the uni-modality approach, each data modality was processed separately without incorporating cross-modal interactions, and the learnable representations were then passed to a classifier for final prediction. The pure multi-modality fusion approach, by contrast, utilized only the joint latent representation for final prediction, omitting any modality-specific features. The final results are shown in Table 2. The specific calibration diagrams and decision curve analysis are shown in Figures S2 and S3. The results showed that MULTINet, which integrated both unimodal and multimodal learning, significantly outperformed methods relying solely on individual modalities or those using only modality fusion, demonstrating superior overall performance.

A systematic ablation experiment was conducted on the key structure and design of the model, and the results are shown in Table 2. The specific calibration diagrams and decision curve analysis are shown in Figures S4–S6. Firstly, the differences between parameter freezing and fine-tuning on the ECG-FM model were compared. The experimental results show that fine-tuning the ECG-FM model can further slightly improve the overall prediction performance, but it also brings higher computational overhead at the same time. Secondly, in the comparison between attention pooling and mean pooling, the attention pooling mechanism proposed in this study performs more prominently in multimodal information fusion and can effectively enhance the prediction effect of the model. Finally, in the data preprocessing stage, using the MICE algorithm for missing value interpolation is significantly superior to simply adopting the median interpolation method, further demonstrating the positive impact of a reasonable data processing strategy on model performance.

The performance of MULTINet in subgroups of different genders and ages was further evaluated, and the results are shown in Table 3. Most patients receiving TAVR were elderly, with a median age of 82 years (SD: 9.33). Studies have shown that patients over 80 years old have an increased risk of readmission, complications and mortality [28]. Therefore, patients were stratified by age for subgroup analysis to compare the prognosis of those aged ≥ 80 years with those aged < 80 years. In different age subgroups, the predictive effect of patients < 80 years was higher than that of those ≥ 80 years. In terms of gender, the results showed that the predictive performance of men and women was balanced.

**Figure 3:**
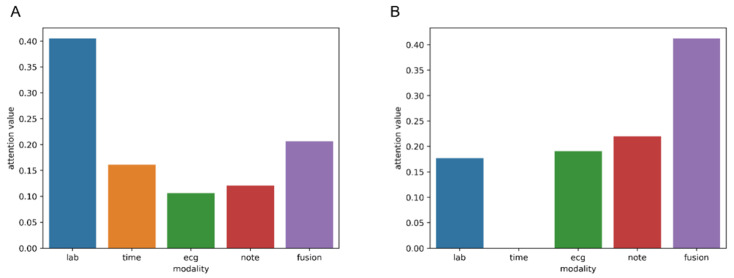
Attention scores of different modalities: (**A**) Full-modal data input result. (**B**) Missing time series modal input result. lab: laboratory tests; time: time series; note: text annotation; ecg: electrocardiogram; fusion: learnable query embedding; note: clinical text annotations.

The attention pooling fusion module can adaptively handle the final fused inputs from different modalities and, in cases where certain modal inputs are missing, automatically down-weight or ignore the corresponding modality while amplifying the contributions of the available modalities. To effectively clarify the role of the attention pooling fusion module, input samples with normal and missing modalities were randomly selected, and the attention scores of different inputs were visualized, as shown in Figure 3. The results demonstrated that the attention pooling fusion module achieved adaptive score allocation across different modal inputs. When all modalities were present (Figure 3A), the model assigned the highest weight to lab, followed by fusion, suggesting that lab results played a major role in this sample prediction. However, in the case of missing modalities, such as when the patient did not enter the ICU (Figure 3B), namely, when the time-series data modality of the ICU was missing, the scores of this modality input were reset to 0, and only the existing other modality information inputs were considered for the final decision. The result indicated that the proposed method not only highlighted the contribution of key modality but also achieved dynamic compensation when modality was missing, thereby enhancing the robustness and reliability of the decision-making process.

**Figure 4:**
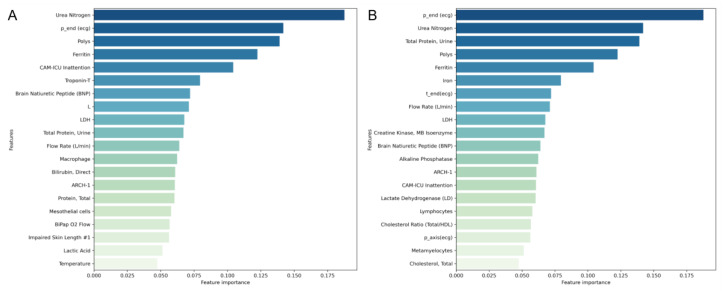
Feature importance ranking: (**A**) The ranking of the average feature importance of all short-term deceased patients. (**B**) The ranking of the feature importance of individual ICU emergency patients.

To investigate the key indicators for predicting high-risk patients, the IG algorithm was used to calculate the average importance of input features for all patients with death in the short term, and the top 20 important features were plotted in Figure 4A. Owing to patient heterogeneity, there were also partial differences in the importance of specific features for each patient. Therefore, a critically ill patient admitted to the ICU was randomly selected, and its top 20 important features were also plotted and compared with the overall results in Figure 4B. The aggregated feature-importance analysis revealed that renal function, systemic inflammation/immune status, and cardiac condition were the domains with the highest overall importance. In particular, Urea Nitrogen, P-wave end point, Polymorphonuclear leukocytes, and troponin-T emerged as highly important features. At the individual level, the feature-importance ranking corroborated these findings: renal function markers, myocardial injury indicators, and measurements of systemic inflammatory or immune imbalance were among the top contributors. Moreover, since the selected case was an emergency ICU admission, ventilator flow rate appeared prominently, reflecting both the severity of respiratory compromise and its close association with mortality risk. The patterns of attribution were generally consistent across subgroups stratified by age and sex, as shown in Figures S8 and S9. Features related to renal function, systemic inflammation, and ECG-derived features repeatedly appeared among the major contributors to model predictions. In contrast, troponin-T showed significant subgroup variability, ranking higher only in the female subgroup. And Brain Natriuretic Peptide (BNP) contributed strongly in multiple subgroups. Because the majority of patients were elderly, in the subgroup of elderly patients, the importance of features as shown in Figure S9 indicated that the CAM-ICU (Confusion Assessment Method for the Intensive Care Unit) Inattention score also stood out as a critical indicator.

## 4. Discussion

### 4.1. Principal Findings

In this study, a 30-day all-cause mortality prediction model was constructed using the clinical multimodal data of patients who underwent TAVR surgery in MIMIC-IV. Our research is in line with the current macrotrend of AI application in cardiovascular electrophysiology. AI has been used to promote individualized cardiology management from multiple dimensions such as ECG interpretation, intraoperative navigation, and risk stratification [29]. Based on this, we further integrated multimodal data, including original ECG waveforms, ICU time series records and clinical texts, to construct a more comprehensive risk prediction model. To our knowledge, MULTINet is one of the first frameworks to integrate raw multimodal such as preoperative laboratory tests, ICU time series monitoring, electrocardiograms, and clinical diagnosis and treatment texts through a unified learnable latent fusion module, as well as to achieve dynamic compensation in missing data patterns by using learnable latent feature representations.

Machine learning studies rely heavily on structured tabular features or a small number of features extracted from images [8,9,10,30,31], the use of raw multimodal inputs remains uncommon in the context of post-procedural risk prediction for TAVR. And the previous studies reported AUC scores mostly in the range of 0.7 to 0.8, which is of limited value in postoperative risk prediction. Agasthi et al. [9] reported an AUC of approximately 0.72 when clinical, laboratory, CT, ECG, and echocardiographic features were fed into a GBM model to predict all-cause mortality at 1 year after surgery. Similarly, Tomii et al. [32] used manual feature extraction including clinical, laboratory, CT, ECG, echocardiography, and surgical operations, combined with random forest/other machine learning methods to predict the technical failure of TAVR. The reported AUC was in the range of approximately 0.77 to 0.79. These method models focus on modeling the relationships of these structured variables and do not fully utilize the fine-grained spatio-temporal signals in the original images/waveforms and other modalities or the complex interactions between modalities. Although machine learning has been widely applied in TAVR risk prediction, research on end-to-end deep learning and true primitive multimodal fusion remains relatively scarce.

In this work, an end-to-end deep learning approach was adopted to automatically capture fine-grained clinically relevant signals that were difficult to encode through manual feature engineering. By jointly modeling complex cross-modal interactions, the proposed method achieved substantially improved predictive performance, with higher discrimination and calibration compared to conventional methods. This study takes the prediction of all-cause mortality after TAVR as an example to reveal the effectiveness of the MULTINet method in integrating multimodal diagnosis and treatment information and hopes to further extend it to the prediction of postoperative mortality risk for other cardiovascular diseases.

### 4.2. Missing Modality Problem

Patients at different stages of the disease course may exhibit specific modal deficiency patterns due to differences in the diagnosis and treatment processes. For instance, critically ill patients may be unable to complete some examinations due to rapid disease progression, while those with milder conditions may lack continuous monitoring data. Previous studies employing traditional machine learning algorithms typically rely on fixed-dimensional and fully aligned input feature vectors, often exclude patients with missing data during the experimental design phase, or implicitly assume the availability of all modalities. This “complete data” assumption leads to an overestimation of model generalizability in real-world clinical settings, where data incompleteness is prevalent, thereby significantly limiting its practical utility in routine care. By introducing the attention pooling fusion module and learnable latent feature representations proposed herein, the model adaptively integrated unimodal and multimodal information according to each patient’s data characteristics, even if some modalities were missing. Notably, Figure 3 shows that, in the case of modal absence, the latent-feature representation received higher attention weights, indicating that the model effectively leveraged fused modalities to compensate for missing information and thereby enhanced robustness and generalizability in real-world applications.

### 4.3. Model Interpretability

In clinical practice, model interpretability is critical in determining acceptance of artificial intelligence by the medical staff. Effective interpretability not only enhances the transparency of model predictions but also improves the understanding and trust of the medical staff [33], further helping doctors identify potential risk signals and thereby improving the quality of clinical decision making [34]. The IG method attributes the contributions of different input features based on the model’s predictions. By ranking overall feature importance, we first plotted the primary contributing features across all patients who died within 30 days and then performed an explainability analysis on a single emergency ICU case to illustrate patient-specific variations. At the overall level, urea nitrogen took the lead in contribution, which is highly consistent with the conclusion that it is an independent predictor of death in TAVR patients with severe aortic stenosis [35]. This result was consistent across different age and gender subgroups, as shown in Figures S8 and S9. Electrocardiogram-related features [36], Brain Natriuretic Peptide (BNP) [37], and troponin T [38] were also highly ranked, reflecting underlying atrial conduction burden and myocardial injury [39], which have been shown to elevate 30-day to 1-year mortality risk [40]. Markers of inflammation and oxygen consumption such as the serum ferritin, lactate dehydrogenase and macrophages collectively indicated systemic inflammation and immune activation, and multiple studies have confirmed their association with heart failure, rehospitalization and one-year mortality [41,42,43]. Furthermore, since valvular diseases predominantly affect elderly patients, as shown in Figure S9, the CAM-ICU inattention score highlighted the impact of delirium on postoperative mortality risk in this frail population [44]. In the individual-level feature importance ranking, the highest-ranked indicators again involved myocardial function, renal function, and systemic inflammation and oxygen-metabolism markers, which were basically consistent with the overall results. It is important to recognize that, although the IG method offers meaningful feature contributions by quantifying the sensitivity of the model output to input features, these contributions capture statistical associations rather than causal effects, reflecting a broader challenge in artificial intelligence for healthcare.

### 4.4. Limitations and Future Directions

This study has made certain advancements in methodological exploration and model development, yet several limitations remain. Owing to practical challenges in the acquisition, integration, and storage of multimodal clinical data, the data sources employed here are relatively homogeneous, relying solely on the publicly available MIMIC-IV dataset for model training and evaluation. This limitation in data provenance may impair the model’s generalizability and external validity, particularly when applied to real-world settings characterized by diverse patient populations, heterogeneity in data acquisition devices, and variations in clinical workflows. Moreover, varying degrees of missingness in the multimodal data constrain our ability to comprehensively model the interactions among different information sources, which may affect both predictive performance and the depth of interpretability. The 30-day mortality rate in the TAVR cohort of this study was only 4.2%, and the positive samples were scarce, which easily led to the model learning noise or accidental associations, increasing the risk of overfitting and prediction uncertainty. Despite the use of 5-fold cross-validation and bootstrapping methods for stable evaluation, a low event rate still amplifies the variance of performance indicators, which may lead to overly optimistic results. In addition, simultaneous or combined surgeries may pose potential clinical confusion among the influencing factors predicted by the model. In the Table S1 results of this study, the performance of the model showed only limited differences before and after including the information related to parallel surgeries, indicating that the model has certain robustness against such confounding factors. There may be other factors in clinical practice that have not been fully observed or fully encoded, such as intraoperative management strategies and perioperative treatment approaches. Therefore, how to more systematically identify and control multi-dimensional potential confusions remains an important research direction. Furthermore, this study does not incorporate echocardiography data, which is a cornerstone in the diagnosis and severity assessment of aortic stenosis, as transthoracic echocardiography enables accurate measurement of key hemodynamic parameters—such as aortic valve area (AVA), peak jet velocity, and mean pressure gradient—that are essential for clinical decision making [45]. Future studies may focus on model optimization and multicenter validation across a broader range of datasets to further enhance the practical utility and clinical translatability of the proposed approach.

## 5. Conclusions

In this study, a deep learning model named MULTINet is proposed to predict 30-day all-cause mortality after TAVR with more accurate predictions compared with traditional models. And, combined with interpretable analysis, it reveals that renal function, cardiac function and inflammation-related indicators have a high contribution in the prediction process. This methodological exploration provides clinicians with potential decision-support insights and is expected to be promoted further in a broader range of clinical practice to facilitate precision medicine.

## Figures and Tables

**Table 1:** The performance of different models on multimodal data *.

Method	AUC	AUPR	Recall	Brier
XGBoost	0.8958 (0.85–0.93)	0.4053 (0.26–0.57)	0.7793 (0.62–0.90)	0.0343 (0.03–0.04)
MedFuse	0.5571 (0.53–0.61)	0.2487 (0.15–0.32)	0.3089 (0.15–0.47)	0.2496 (0.24–0.25)
MULTINet	0.9153 (0.87–0.95)	0.5708 (0.48–0.66)	0.8051 (0.71–0.90)	0.0269 (0.02–0.03)

*: Point estimate and 95% CI (in the parentheses) in the table.

**Table 2:** The ablation performance of the preprocessing or designs *.

Method	AUC	AUPR	Recall	Brier
Median interpolation	0.7242 (0.62–0.81)	0.2517 (0.11–0.41)	0.6586 (0.50–0.81)	0.0377 (0.03–0.04)
Uni-modality	0.9015 (0.85–0.94)	0.5553 (0.39–0.70)	0.7491 (0.59–0.89)	0.0329 (0.03–0.04)
Multi-modality	0.8715 (0.86–0.88)	0.4192(0.31–0.50)	0.6845 (0.53–0.83)	0.0389 (0.03–0.04
Mean pooling	0.8977 (0.84–0.94)	0.4385 (0.28–0.61)	0.7790 (0.63–0.90)	0.0328 (0.03–0.04)
ECG-FM finetuned	0.9286 (0.88–0.96)	0.5325 (0.38–0.70)	0.8463 (0.71–0.96)	0.0287 (0.02–0.03)
MULTINet	0.9153 (0.87–0.95)	0.5708 (0.48–0.66)	0.8051 (0.71–0.90)	0.0269 (0.02–0.03)

*: Point estimate and 95% CI (in the parentheses) in the table.

**Table 3:** The performance of different subgroups *.

	AUC	AUPR	Recall	Brier
Age				
<80 (307, 40%)	0.8808 (0.78–0.96)	0.5907 (0.52–0.64)	0.8165 (0.62–0.98)	0.0268 (0.02–0.03)
≥80 (454, 60%)	0.8779 (0.76–0.97)	0.5565 (0.55–0.56)	0.8541 (0.64–0.99)	0.0269 (0.02–0.03)
Sex				
Male (402, 53%)	0.8812 (0.83–0.93)	0.5108 (0.37–0.65)	0.8208 (0.72–0.92)	0.0361 (0.03–0.04)
Female (359, 47%)	0.8803 (0.80–0.94)	0.5715(0.36–0.78)	0.8013 (0.78–0.82)	0.0336 (0.03–0.04)

*: Point estimate and 95% CI (in the parentheses) in the table.

## Data Availability

The data used in this study can be accessed on https://physionet.org/content/mimiciv/3.0/ (accessed on 27 December 2024) by registering and submitting a request. The model weights of the ECG-FM model used are from the public version released by Wang Lab (https://huggingface.co/wanglab/ecg-fm). Accessed and downloaded on 10 November 2025. The model weights of the Clinical ModernBERT model used are from the public version released by Simonlee711 (https://huggingface.co/Simonlee711/Clinical_ModernBERT). Accessed and downloaded on 10 November 2025.

## Supplementary Materials

The following supporting information can be downloaded at: https://www.mdpi.com/article/10.3390/jcm14248620/s1.

## Abbreviations

The following abbreviations are used in this manuscript:

SD	Standard deviation
TAVR	Transcatheter aortic valve replacement
MULTINet	Multimodal learning for TAVR risk network
ICU	Intensive care unit
MIMIC-IV	Medical Information Mart for Intensive Care IV
AUC	Area under the receiver operating characteristic curve
AUPR	Area under the Precision–Recall curve
IGs	Integrated gradients
XGBoost	eXtreme Gradient Boosting
EuroSCORE II	European System for Cardiac Operative Risk Evaluation II
PROM	Society of Thoracic Surgeons Predicted Risk of Mortality
ECGs	Electrocardiograms
ICD	International classification of diseases
MICE	Multiple imputation by chained equations
LOCF	Last observation carried forward
CT	Computed tomography
MRI	Magnetic resonance imaging
ECG-FM	Electrocardiogram foundation model
M-MHSA	masked multi-head self-attention
M-MHCA	masked multi-head cross-attention
DFL	dual focal loss
FP	false positive
FN	False Negative
TP	true positive
TN	true negative
FPR	false positive rate
TPR	true positive rate
HAIM	Holistic AI in Medicine
LSTM	long short-term memory
CAM-ICU	confusion assessment method for the intensive care unit
BNP	brain natriuretic peptide
UN	urea nitrogen
AVA	aortic valve area

## References

[1] Kumar V., Sandhu G.S., Harper C.M., Ting H.H., Rihal C.S. (2020). Transcatheter aortic valve replacement programs: Clinical outcomes and developments. J. Am. Heart Assoc..

[2] Vinayak M., Leone P.P., Tanner R., Dhulipala V., Camaj A., Makhija R.R.K., Hooda A., Kini A.S., Sharma S.K., Khera S. (2024). Transcatheter aortic valve replacement: Current status and future indications. J. Clin. Med..

[3] Lim G.B. (2023). Suitability of TAVI in low-risk patients. Nat. Rev. Cardiol..

[4] Puskas J.D., Kilgo P.D., Thourani V.H., Lattouf O.M., Chen E., Vega J.D., Cooper W., Guyton R.A., Halkos M. (2012). The society of thoracic surgeons 30-day predicted risk of mortality score also predicts long-term survival. Ann. Thorac. Surg. Off. J. Soc. Thorac. Surg. South. Thorac. Surg. Assoc..

[5] Nashef S.A.M., Roques F., Sharples L.D., Nilsson J., Smith C., Goldstone A.R., Lockowandt U. (2012). EuroSCORE II. Eur. J. Cardio-Thorac. Surg..

[6] Cao X., Wang C., Wang Q., Li X., Han L., Xu Z., Zou L. (2011). Validation of the EuroSCORE and the STS-PROM in adult patients undergoing aortic valve replacement. Chin. J. Thorac. Cardiovaescular Surg..

[7] Lopes R.R., van Mourik M.S., Schaft E.V., Ramos L.A., Baan J., Vendrik J., de Mol B.A.J.M., Vis M.M., Marquering H.A. (2019). Value of machine learning in predicting TAVI outcomes. Neth. Heart J..

[8] Penso M., Pepi M., Fusini L., Muratori M., Cefalù C., Mantegazza V., Gripari P., Ali S.G., Fabbiocchi F., Bartorelli A.L. (2021). Predicting long-term mortality in TAVI patients using machine learning techniques. J. Cardiovasc. Dev. Dis..

[9] Agasthi P., Ashraf H., Pujari S.H., Girardo M.E., Tseng A., Mookadam F., Venepally N.R., Buras M., Khetarpal B.K., Allam M. (2021). Artificial intelligence trumps TAVI2-SCORE and CoreValve score in predicting 1-year mortality post-transcatheter aortic valve replacement. Cardiovasc. Revascularization Med..

[10] Kwiecinski J., Dabrowski M., Nombela-Franco L., Grodecki K., Pieszko K., Chmielak Z., Pylko A., Hennessey B., Kalinczuk L., Tirado-Conte G. (2023). Machine learning for prediction of all-cause mortality after transcatheter aortic valve implantation. Eur. Heart J.-Qual. Care Clin. Outcomes.

[11] Gomes B., Pilz M., Reich C., Leuschner F., Konstandin M., Katus H.A., Meder B. (2021). Machine learning-based risk prediction of intrahospital clinical outcomes in patients undergoing TAVI. Clin. Res. Cardiol..

[12] Huang S.-C., Pareek A., Seyyedi S., Banerjee I., Lungren M.P. (2020). Fusion of medical imaging and electronic health records using deep learning: A systematic review and implementation guidelines. NPJ Digit. Med..

[13] Soenksen L.R., Ma Y., Zeng C., Boussioux L., Carballo K.V., Na L., Wiberg H.M., Li M.L., Fuentes I., Bertsimas D. (2022). Integrated multimodal artificial intelligence framework for healthcare applications. NPJ Digit. Med..

[14] Le L.P., Nguyen T., Riegler M.A., Halvorsen P., Nguyen B.T. (2025). Multimodal missing data in healthcare: A comprehensive review and future directions. Comput. Sci. Rev..

[15] Acosta J.N., Falcone G.J., Rajpurkar P., Topol E.J. (2022). Multimodal biomedical AI. Nat. Med..

[16] Mamprin M., Zelis J.M., Tonino P.A.L., Zinger S., de With P.H.N. (2021). Decision trees for predicting mortality in transcatheter aortic valve implantation. Bioengineering.

[17] Yu Y., Li M., Liu L., Li Y., Wang J. (2019). Clinical big data and deep learning: Applications, challenges, and future outlooks. Big Data Min. Anal..

[18] Johnson A.E.W., Bulgarelli L., Shen L., Gayles A., Shammout A., Horng S., Pollard T.J., Hao S., Moody B., Gow B. (2023). MIMIC-IV, a freely accessible electronic health record dataset. Sci. Data.

[19] Van Buuren S., Groothuis-Oudshoorn K. (2011). mice: Multivariate imputation by chained equations in R. J. Stat. Softw..

[20] Sugden R.A. (1988). Multiple Imputation for Nonresponse in Surveys. J. R. Stat. Soc. Ser. A Stat. Soc..

[21] Harel O., Mitchell E.M., Perkins N.J., Cole S.R., Tchetgen Tchetgen E.J., Sun B., Schisterman E. (2018). Multiple imputation for incomplete data in epidemiologic studies. Am. J. Epidemiol..

[22] McKeen K., Oliva L., Masood S., Rubin B., Wang B. (2024). Ecg-fm: An open electrocardiogram foundation model. arXiv.

[23] Lee S.A., Wu A., Chiang J.N. (2025). Clinical modernbert: An efficient and long context encoder for biomedical text. arXiv.

[24] Lin T.Y., Goyal P., Girshick R., He K., Dollár P. Focal loss for dense object detection. Proceedings of the IEEE International Conference on Computer Vision.

[25] Tao L., Dong M., Xu C. Dual focal loss for calibration. Proceedings of the International Conference on Machine Learning, ICML.

[26] Sundararajan M., Taly A., Yan Q. Axiomatic attribution for deep networks. Proceedings of the International Conference on Machine Learning, ICML.

[27] Hayat N., Geras K.J., Shamout F.E. MedFuse: Multi-modal fusion with clinical time-series data and chest X-ray images. Proceedings of the Machine Learning for Healthcare Conference, MLHC.

[28] Delijani D., Li L., Rutkin B., Wilson S., Kennedy K.F., Hartman A.R., Yu P.J. (2023). Impact of age on outcomes after transcatheter aortic valve implantation. Eur. Heart J. Qual. Care Clin. Outcomes.

[29] Cersosimo A., Zito E., Pierucci N., Matteucci A., La Fazia V.M. (2025). A Talk with ChatGPT: The Role of Artificial Intelligence in Shaping the Future of Cardiology and Electrophysiology. J. Pers. Med..

[30] Alhwiti T., Aldrugh S., Megahed F.M. (2023). Predicting in-hospital mortality after transcatheter aortic valve replacement using administrative data and machine learning. Sci. Rep..

[31] Kwiecinski J., Grodecki K., Pieszko K., Dabrowski M., Chmielak Z., Wojakowski W., Niemierko J., Fijalkowska J., Jagielak D., Ruile P. (2025). Preprocedural CT angiography and machine learning for mortality prediction after transcatheter aortic valve replacement. Prog. Cardiovasc. Dis..

[32] Tomii D., Shiri I., Baj G., Nakase M., Kazaj P.M., Samim D., Bartkowiak J., Praz F., Lanz J., Stortecky S. (2025). Multimodal Machine Learning-Based Technical Failure Prediction in Patients Undergoing Transcatheter Aortic Valve Replacement. JACC Adv..

[33] Ghassemi M., Oakden-Rayner L., Beam A.L. (2021). The false hope of current approaches to explainable artificial intelligence in health care. Lancet Digit. Health.

[34] Tomsett R., Preece A., Braines D., Cerutti F., Chakraborty S., Srivastava M., Pearson G., Kaplan L. (2020). Rapid trust calibration through interpretable and uncertainty-aware AI. Patterns.

[35] Haberman D., Shimoni S., George J. (2017). Blood Urea Predicts All-cause Mortality in Patients with Severe Aortic Stenosis. Circulation.

[36] Gulsen K., Ince O., Akgun T., Demir S., Uslu A., Kup A., Ocal L., Emiroglu M.Y., Kargin R., Sahin I. (2020). The effect of P wave indices on new onset atrial fibrillation after trans-catheter aortic valve replacement. J. Electrocardiol..

[37] Lehtola H., Piuhola J., Niemelä M., Tauriainen T., Junttila J., Mäkikallio T., Juvonen T., Biancari F. (2022). B-type natriuretic peptide ability to predict mortality after transcatheter aortic valve replacement. J. Cardiovasc. Med..

[38] Seoudy H., Lambers M., Winkler V., Dudlik L., Freitag-Wolf S., Frank J., Kuhn C., Rangrez A.Y., Puehler T., Lutter G. (2021). Elevated high-sensitivity troponin T levels at 1-year follow-up are associated with increased long-term mortality after TAVR. Clin. Res. Cardiol..

[39] Imamura T., Oshima A., Narang N., Onoda H., Tanaka S., Ushijima R., Sobajima M., Fukuda N., Ueno H., Kinugawa K. (2022). Clinical implications of troponin-T elevations following TAVR: Troponin Increase Following TAVR. J. Cardiol..

[40] Schindler M., Stöckli F., Brütsch R., Jakob P., Holy E., Michel J., Manka R., Vogt P., Templin C., Kasel M. (2021). Postprocedural troponin elevation and mortality after transcatheter aortic valve implantation. J. Am. Heart Assoc..

[41] Khalil C., Pham M., Sawant A.C., Sinibaldi E., Bhardwaj A., Ramanan T., Qureshi R., Khan S., Ibrahim A., Gowda S.N. (2018). Neutrophil-to-lymphocyte ratio predicts heart failure readmissions and outcomes in patients undergoing transcatheter aortic valve replacement. Indian Heart J..

[42] Rawat A., Goyal P., Ahsan S.A., Srivyshnavi K.S.S., Asghar A.H., A Riyalat A., Wei C.R., Khan A. (2025). The Prognostic Value of Neutrophil-to-Lymphocyte Ratio on Mortality in Patients Undergoing Transcatheter Aortic Valve Implantation: A Systematic Review and Meta-Analysis. Cureus.

[43] Hoffmann J., Mas-Peiro S., Berkowitsch A., Boeckling F., Rasper T., Pieszko K., De Rosa R., Hiczkiewicz J., Burchardt P., Fichtlscherer S. (2020). Inflammatory signatures are associated with increased mortality after transfemoral transcatheter aortic valve implantation. ESC Heart Fail..

[44] Prasitlumkum N., Mekritthikrai R., Kewcharoen J., Kanitsoraphan C., Mao M.A., Cheungpasitporn W. (2020). Delirium is associated with higher mortality in transcatheter aortic valve replacement: Systemic review and meta-analysis. Cardiovasc. Interv. Ther..

[45] Caldararu C., Balanescu S. (2016). Modern Use of Echocardiography in Transcatheter Aortic Valve Replacement: An Up-Date. Maedica.

