# Supplementary material for: The Application of Multimodal Data Fusion Algorithm MULTINet in Postoperative Risk Assessment of TAVR
Source: J Clin Med. 2025 Dec 5;14(24):8620. doi: 10.3390/jcm14248620 (PMC12734006; doi:10.3390/jcm14248620)
Supplement: Supplementary file 1 [file jcm-14-08620-s001.zip › jcm-3990254-supplementary.pdf]

Journal of
Clinical Medicine

MDPI

Supplementary Materials

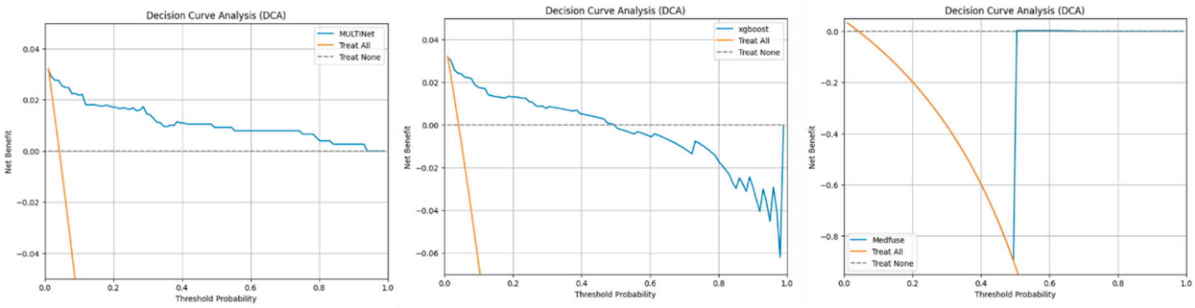

Figure S1 Detail decision curve analysis results of different models

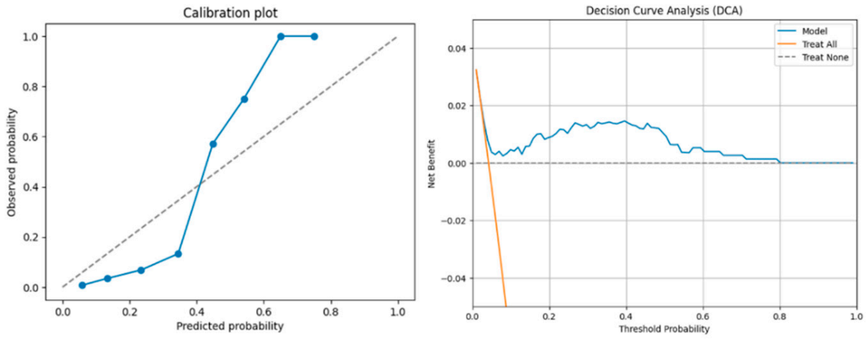

Figure S2 Uni-modality calibration and decision curve analysis results

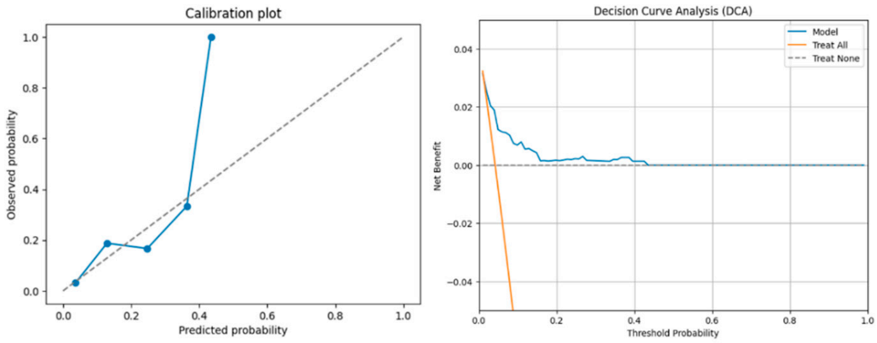

Figure S3 Multi-modality calibration and decision curve analysis results

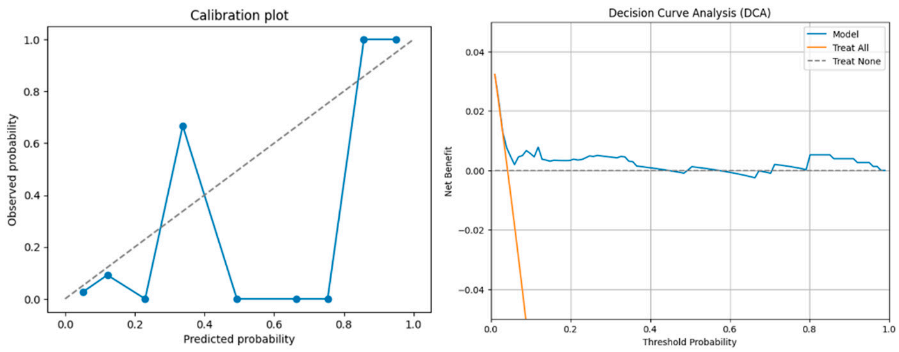

Figure S4 Median interpolation calibration and decision curve analysis results

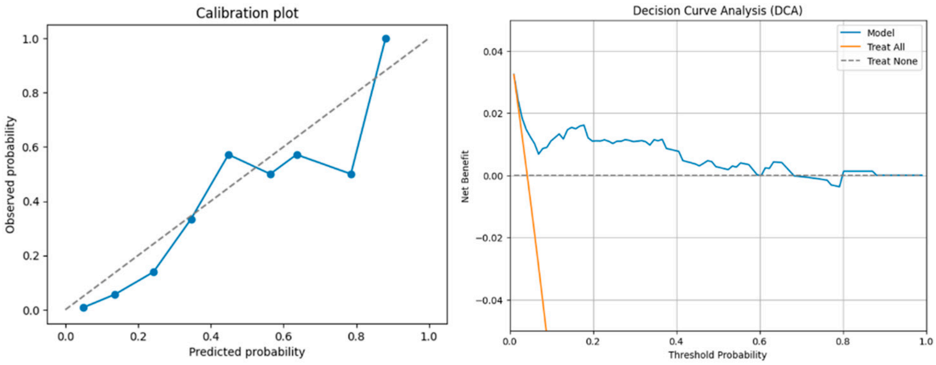

Figure S5 Mean pooling calibration and decision curve analysis results

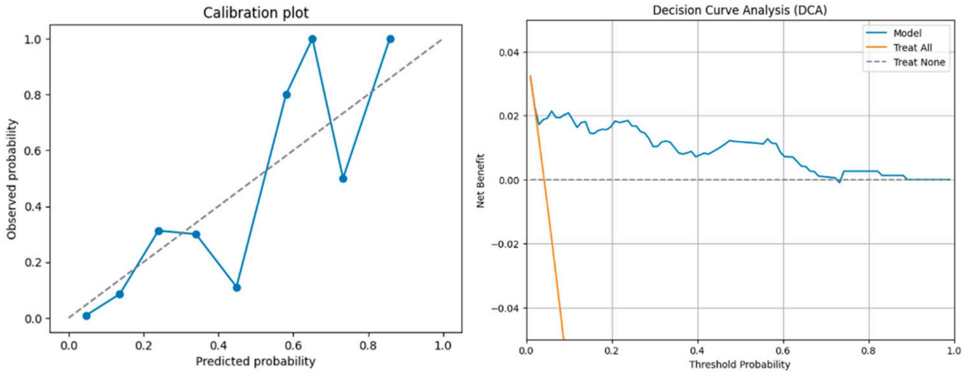

Figure S6 ECG pretrain model finetuned calibration and decision curve analysis results

For the potential confounding variables that may be introduced due to concurrent or combined surgeries, we conducted additional analysis. We included the descriptions of relevant surgical operations admitted during the same period as TAVR from the surgical procedures as text modalities, enabling the model to explicitly handle the existence of combined intervention measures. This strategy can reduce residual confusion and allow the model to directly learn program-related context from clinical texts. The results are shown in Table S1. The corresponding calibration diagrams and decision analysis are in Figure S4. The results show that increasing the data will lead to a slight decline in model performance, indicating that combined surgery does indeed affect model performance.

Table S1 Add the description of the combined surgery

Method	AUC	AUPR	Recall	Brier
Procedure	0.9151 (0.86-0.96)	0.5519 (0.38-0.70)	0.8744 (0.75-0.96)	0.0317 (0.03-0.04)
MULTINet	0.9153 (0.87-0.95)	0.5708 (0.48-0.66)	0.8051 (0.71-0.90)	0.0269 (0.02-0.03)

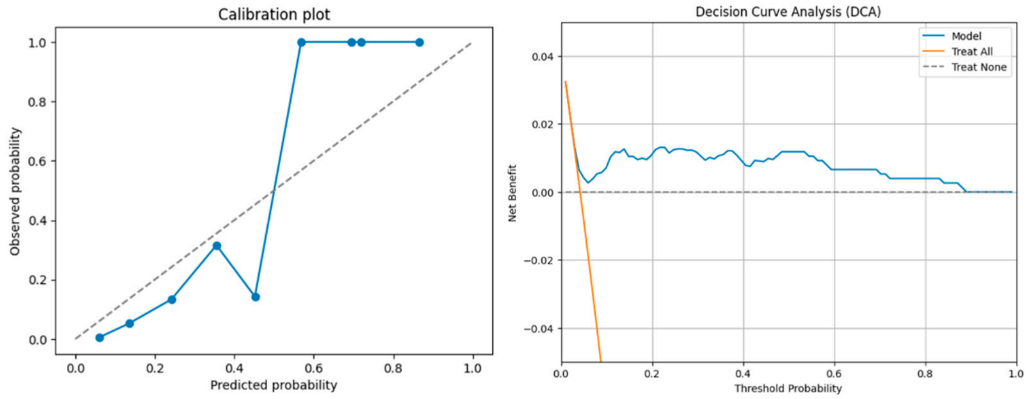

Figure S7 Add the description of the combined surgery calibration and decision curve analysis results

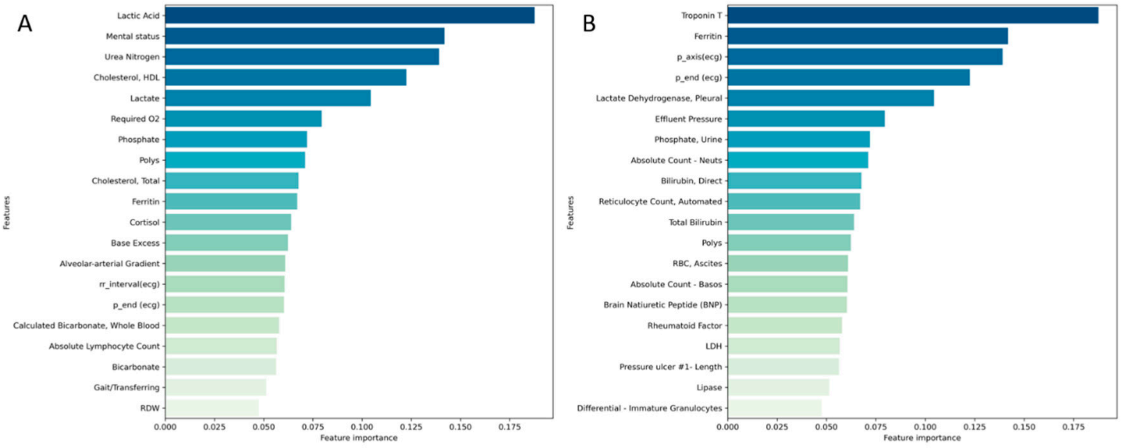

Figure S8 Feature importance ranking. A: The ranking of the average feature importance of female subgroup patients. B: The ranking of the average feature importance of male subgroup patients.

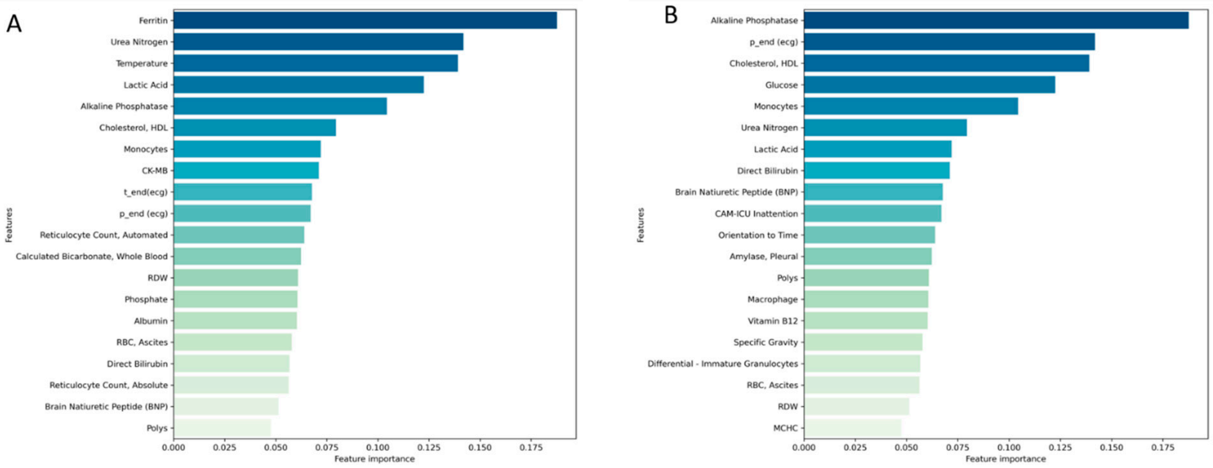

Figure S9 Feature importance ranking. A: The ranking of the average feature importance of <80 years subgroup patients. B: The ranking of the average feature importance of ⩾80 years subgroup patients.

Table S2 The screened ICD code

icd_code	icd_version
3505	9
3506	9
02RF37H	10
02RF37Z	10
02RF38H	10
02RF38Z	10
02RF3JH	10
02RF3JZ	10
02RF3KH	10
02RF3KZ	10
02RF47Z	10
02RF48Z	10
02RF4JZ	10
02RF4KZ	10
X2RF332	10
X2RF432	10
